# Pequi (*Caryocar brasiliense*) Waste Extract as a Synergistic Agent in the Microbial and Physicochemical Preservation of Low-Sodium Raw Goat Cheese

**DOI:** 10.3389/fnut.2022.855115

**Published:** 2022-04-06

**Authors:** Rodrigo V. Moreira, Carla P. Vieira, Diego Galvan, Vinicius S. Castro, Rayssa S. Lima, Yhan S. Mutz, Karina F. Delgado, Anisio Iuri L. Rosario, Sérgio B. Mano, Marion P. Costa, Carlos A. Conte-Junior

**Affiliations:** ^1^Graduate Program in Veterinary Hygiene (PPGHV), Faculty of Veterinary Medicine, Fluminense Federal University (UFF), Rio de Janeiro, Brazil; ^2^Center for Food Analysis (NAL), Technological Development Support Laboratory (LADETEC), Federal University of Rio de Janeiro (UFRJ), Rio de Janeiro, Brazil; ^3^Laboratory of Advanced Analysis in Biochemistry and Molecular Biology (LAABBM), Department of Biochemistry, Federal University of Rio de Janeiro (UFRJ), Rio de Janeiro, Brazil; ^4^Graduate Program in Food Science (PPGCAL), Institute of Chemistry (IQ), UFRJ, Rio de Janeiro, Brazil; ^5^Laboratory of Inspection and Technology of Milk and Derivatives, Faculty of Veterinary Medicine, Federal University of Bahia, Salvador, Brazil; ^6^Graduate Program in Chemistry (PGQu), Institute of Chemistry (IQ), Federal University of Rio de Janeiro (UFRJ), Rio de Janeiro, Brazil; ^7^Graduate Program in Sanitary Surveillance (PPGVS), National Institute of Health Quality Control (INCQS), Oswaldo Cruz Foundation (FIOCRUZ), Rio de Janeiro, Brazil

**Keywords:** non-thermal technologies, sodium reduction, pathogenic microorganisms, raw milk cheese, cheese preservation

## Abstract

The growth of spoilage and pathogenic bacteria during storage represents significant losses in marketing raw milk cheeses. Thus, reducing NaCl in these products is challenging, as sodium has a critical antimicrobial role. Despite advances in non-thermal technologies, the short shelf life still limits the availability of raw goat cheese. Thus, combined preservation methods can be promising because their synergies can extend shelf life more effectively. In this context, Principal Component Analysis (PCA) was applied to variables to investigate the effect of pequi waste extract (PWE), a native Brazilian fruit, combined with UV-C radiation (CEU) and vacuum packaging (CEV) on the preservation of low-sodium raw goat cheese. CEV samples had lower loadings for *Staphylococcus* subsp. and *Enterobacteriaceae* than other treatments in PC2, having a count’s reduction up to 3-fold (*P* < 0.05) compared to vacuum alone. In contrast, CEU showed an increase of up to 1.2-fold on staphylococcal count compared to UV-C alone. Still, the addition of PWE to UV-C-treated cheeses resulted in 8.5% protein loss. Furthermore, PWE, especially in CEV, delayed post-acidification during storage. It made CEV up to 4.5 and 1.6-fold more stable for color and texture, respectively than vacuum alone. These data strongly suggest that PWE may be a novel and promising synergistic agent in the microbial and physicochemical preservation of low-sodium raw milk cheese when combined with the vacuum.

## Introduction

Cheese is a nutrient-dense dairy product and, consequently, susceptible to physical, chemical, and biochemical spoilage (1). However, microbial contamination, in addition to interfering with food quality, reducing the product’s shelf life, can also compromise food safety due to the presence of pathogens (2). Raw cheeses are those produced with unpasteurized or heat untreated milk. Milk pasteurization commonly used in modern industrial cheese production aims to eliminate pathogens and spoilage bacteria. Nevertheless, artisanal cheesemakers may still use raw milk (3). Raw milk cheeses present social and economic importance, especially in the European Union and Switzerland, due to their large-scale production and widespread production areas (4). In addition, artisanal goat cheeses are considered an excellent source of proteins, lipids, vitamins, and mineral elements (5).

The stability of raw milk cheeses depends mainly on growth control and degradation moderation by spoilage microorganisms (1). The significant losses during the commercialization of raw cheeses occur, especially during the storage, due to the growth of spoilage and potentially pathogenic bacteria arising from raw milk or the dairy environment (6). *Staphylococcus aureus* and *Enterobacteriaceae* as *Salmonella* sp. and *Escherichia coli* (7–9) constitute the primary foodborne pathogens that are usually grown in food such as dairy products (10, 11). Due to these reasons, reducing NaCl in these products is even more challenging, as sodium has a critical antimicrobial role (12), which is not played by KCl (13). Sodium reduction has been proposed as a technological alternative for obtaining healthier dairy products (14).

The addition of chemical preservatives is one of the simple and oldest ways to extend the shelf life of cheese (1). However, these additives are not pleasing to many consumers, generating a strong demand for alternative preservation methods (15). In addition, microorganisms have shown some resistance to chemical preservatives (16). In this context, non-thermal technologies represent the possibility of preserving the cheeses for a longer time and thus increasing their shelf life. Indeed, both Vacuum and UV-C radiation are effective food preservation methods. However, they have some limitations. UV-C radiation only acts on the surface, having low power to penetrate the sample; that is, the interior of the cheeses would be vulnerable to microbial growth (17). Vacuum packaging is a very effective conservation method in delaying the growth of aerobic bacteria through oxygen reduction. However, such a reduction may be favorable for other bacteria present in cheese (anaerobic and microaerophilic) (18). Furthermore, regarding physicochemical parameters, UV-C treatment can lead to free-radical as well as photochemical reactions in food. It can result in impaired texture and changes in color (19). Vacuum packaging, on the other hand, can lead to color alteration during storage (20, 21).

The availability of raw goat cheese is still limited by the product’s short shelf life and the seasonal production of goat milk (22). Artisanal goat cheese distribution is gradually shifting from cheesemakers selling directly to their consumers from their farms to large-scale distribution through the market (23). Consequently, research is essential in increasing shelf-life and promoting raw cheese quality and safety to meet this demand. In this context, the combination of preservation methods can be helpful because their synergies can extend shelf life more effectively (1).

Pequi (*Caryocar brasiliense*) is a native fruit from Brazil whose extract presents antimicrobial activity against spoilage and pathogenic bacteria, mainly attributed to polyphenol, terpenes, and flavonoid compounds (24). Besides, in previous studies performed by our research group, the pequi waste extract (PWE) was demonstrated to be a promising alternative as a preservative method for fresh goat cheese elaborated with pasteurized milk (25, 26). Additionally, pequi waste extract has some advantages regarding UV-C and vacuum treatments, such as penetrating the entire cheese sample since it is added during cheesemaking and has an antimicrobial effect against both anaerobic and aerobic bacteria, as previously shown (27). Therefore, the application of PWE associated with vacuum and UV-C aims to increase the antimicrobial power on the whole cheese and guarantee food safety, especially when considering cheese made from raw milk. In addition, it is interesting to investigate whether pequi extract, which is rich in antioxidants, could contribute to the physicochemical preservation of cheeses in the combined treatments.

In these circumstances, the primary hypothesis of this work was that the addition of PWE acts synergistically with non-thermal methods on the preservation of raw goat milk cheese. Therefore, the objective of this study was to verify the effect of PWE combined with UV-C radiation and vacuum packaging on physicochemical and microbial preservation of low-sodium raw goat cheese over refrigerated storage.

## Materials and Methods

### Pequi Waste Extract

Pequi fruits (*Caryocar brasiliense*) were collected from the Pequi Nino Farm (Montes Claros, Minas Gerais, Brazil) (16° 44′ 06′′ S, 43° 51′ 42′′ W). Pequi epicarps and external mesocarps were processed according to Moreira et al. (25). Briefly, PWE was obtained by microwave-assistant extraction (MAE) in a DGT 100 Plus system (Provecto Analytics Ltd., São Paulo, Brazil) using a 94% (v/v) aqueous ethanol solution and a 670 W microwave power for 110 s. Subsequently, this extract was concentrated in a rota evaporator (Fisatom, model 801) and then diluted to a 6.25 mL/L value. This concentration was defined considering the minimum inhibitory concentration (MIC), previously determined by Paula-Junior et al. (27) as 6.25 and 4 μg/mL for *E. coli* and *Staphylococcus* subsp., respectively.

### Cheesemaking

Raw goat milk (forty litters) was obtained from Sítio Água da Pedra (Niterói, Rio de Janeiro, Brazil) (22° 52′ 58′′ S 43° 06′ 14′′). The unpasteurized milk was added to 0.4 mL/L of 40% (v/v) calcium chloride (Rica Nata), 10 mL/L *Lactococcus lactis* subsp. *lactis* and *Lactococcus lactis* subsp. *cremoris* bacteria (R-704; Chr Hansen, Valinhos, Minas Gerais, Brazil), 6.25 mL/L pequi extract and 1 mL/L liquid coagulant of chymosin (Ha-La^®^; Chr Hansen, Minas Gerais, Brazil). The solution was then mixed for 2 min and left standing for 40 min to coagulate until a firm curd formed. In the next step, the curds were cut gently into 2 cm cubes and allowed to drain. After initial draining was complete, the curd was placed in 250-g perforated circular plastic molds for complete draining after it was turned over twice. Subsequently, the cheeses were dry with 0.8% salt, composed of 75% NaCl (Sigma, São Paulo, Brazil) and 25% KCl (Sigma, São Paulo, Brazil). Finally, the cheeses were packed (aerobic or vacuum packaging), sealed in polyethylene plastic bags, and stored at 4°C for 21 days (25).

Six treatments were performed: (1) raw milk cheese without extract in aerobic packaging (CCA); (2) raw milk cheese without extract in vacuum packaging – VP – (CCV); (3) raw milk cheese without extract in VP + UV-C radiation (CCU); (4) raw milk cheese with PWE in aerobic packaging (CEA); (5) raw milk cheese with PWE in VP (CEV); and, (6) raw milk cheese with PWE in VP + UV-C (CEU).

### UV-C Radiation Exposure

After vacuum packaging, the CCU and CEU treatments were subjected to UV-C radiation in equipment containing six lamps of 30 W and six lamps of 55 W (Osram HNS, Munich, Germany), as designed by Lazaro et al. (28). Before use, the UV lamps were stabilized for 15 min. The intensity levels were monitored using a UV radiometer (MRUR-203, Instrutherm Ltd., São Paulo, Brazil) wrapped with the same sample packaging. The exposure times were measured every 5 s until reaching the dose of 0.1824 ± 0.001 J/cm^2^. This dose was determined by having demonstrated pathogenic bacteria inhibition without gross sensory alteration (29).

### Bacteriological Analysis of the Cheeses

*Lactococcus* subsp*., Staphylococcus* subsp. and *Enterobacteriaceae* counts were performed during the storage period (0, 6, 12, 18, and 21 days) according to American Public Health Association (30). Briefly, 10 g of each cheese was homogenized in 90 mL of 0.1% peptone water in a Stomacher (Stomacher 80, Seward, London, United Kingdom). Then, samples were submitted to serial dilutions and inoculated into Petri dishes using a Spiral Plater (E50, Eddy Jet 2, IUL Instruments, Barcelona, Spain). Enumeration of *Lactococcus* subsp. was performed on M17 agar after incubation under aerobiosis at 35 ± 1°C for 18–24 h. *Enterobacteriaceae* counts were determined through growth on Violet-Red-Bile-Glucose agar (VRBG-agar, Merck, Darmstadt, Germany) and aerobically incubated at 35 ± 1°C for 18–24 h. *Staphylococcus* subsp. were enumerated on Baird-Parker agar (Difco, Detroit, United States) amended with egg yolk-tellurite supplement (Remel, Lenexa, KS, United States) at 35–37 ± 1°C for 18–24 h under aerobiosis. The enumeration of colonies was performed using an electronic counter (Flash & Go, IUL instruments, Barcelona, Spain) after incubation of each bacterium and expressed as log colony forming units (CFU) per gram.

### Physicochemical Analysis

#### Titratable Acidity, pH, and Proximate Composition of Cheese

Moisture, protein, fat, and ash (g/100 g) values were determined in cheese freshly prepared (day 0) following standard procedures (31), being the results expressed as %. Similarly, titratable acidity also was measured according to AOAC (31) and expressed as g/100 g of lactic acid. However, the measurement occurred at the cheese freshly prepared (day 0) and also at the end of storage (21st day).

The pH was verified in 0, 6, 12, 18, and 21 days of storage. Each sample was measured with a digital pH-meter (model PG1800 Cap-Lab Industry and Trade Ltd., São Paulo, Brazil) by direct insertion into the cheese. Before use, the electrode was calibrated with standard buffer solutions of pH 4.00 and 7.00.

#### Instrumental Color

The values of lightness (L*, 100 = white, 0 = black), redness (a*, + red, - green), and yellowness (b*, + yellow, - blue) of the cheeses were recorded at 10°C with a Minolta CM-600D spectrophotometer (Minolta Camera Co., Osaka, Japan) according to Incedayi et al. (32). The color parameters were determined at three random locations on each cheese’s surface in 0, 6, 12, 18, and 21 days of storage immediately after removing the packaging.

The color difference between two samples is often expressed as ΔE (Eq. 1), which describes how visually distant two samples are for color and lightness. The color difference was calculated matching the spectrum of the cheese freshly prepared (*0*) and its relative spectrum at the subsequent storage days (*n*) (33). Thus:


(1) $$\Delta \,E=\sqrt{{\left(L_{n}^{*}-L_{0}^{*}\right)}^{2}+{\left(a_{n}^{*}-a_{0}^{*}\right)}^{2}+{\left(b_{n}^{*}-b_{0}^{*}\right)}^{2}}$$

The difference in the total color saturation of the cheeses during storage was estimated through ΔC* (Eq. 2). It was calculated matching the color saturation of the cheese freshly prepared (*0*) and its relative saturation at the subsequent storage days (*n*) (34), as follows:


(2) Δ⁢C*=(a*)2n+(b*)2n-(a*)20+(b*)20

#### Instrumental Texture Analysis

Hardness (*g*) and cohesiveness (*g*) were measured from the force-deformation curve according to Delgado et al. (35), using a texture analyzer (TA-XT2i, Stable Micro Systems Ltd., Godalming, United Kingdom). It was equipped with a cylinder probe (Aluminum cylinder probe P/36R, 36 mm diameter) and Texture Expert software for Windows (version 1.20; Stable Micro Systems Ltd., Godalming, United Kingdom). The samples were cut into cubes (1 cm × 1 cm × 1 cm) and analyzed at 15 ± 2°C in 0, 6, 12, 18, and 21 days of storage.

### Statistical Analysis

All analyses were performed in analytical and experimental triplicate, and the results were expressed as the mean ± standard deviation (SD). One- and Two-way analysis of variance (ANOVA) was used at a significance level of 0.05, followed by Tukey’s multiple comparison tests (two-side, *P* < 0.05). One-ANOVA was performed to analyze proximate composition data, while Two-ANOVA was used to study texture, color, microbiology, pH, and acidity results. These statistical analyses were performed using a commercially available statistical package (XLSTAT version 2013.2.03 software, Addinsoft, Paris, France).

The linear correlation between variables was evaluated by the Pearson correlation test with a significance level of 0.05. The correlation matrix was performed using the package “corrplot” in the Software R Core Team (Vienna, Austria).

A computational routine implemented in Matlab^®^ 2021a (MathWorks Inc., Natick, MA, United States) was used for pre-processing and multivariate analysis. A Principal Component Analysis – PCA – of the correlation matrix of the *Lactococcus* subsp., *Enterobacteriaceae, Staphylococcus* subsp., instrumental color (L*, a*, and b*), pH, and texture analysis (hardness and cohesiveness) data were performed to investigate behavioral differences between cheeses with and without extract for the different preservation methods tested over time. It was possible to verify the influences between physicochemical and microbiological parameters through the effects of correlations simultaneously concerning the time and treatments evaluated. The scores and loadings were determined using the singular values decomposition (SVD) algorithm (36).

## Results and Discussion

### Effects of Preservative Methods on Bacterial Behavior

Table 1 shows *Lactococcus* subsp., *Enterobacteriaceae* and *Staphylococcus* subsp. counts in raw goat milk cheeses. For *Lactococcus* subsp., in general, there was no difference between treatments with and without extract during storage (*P* > 0.05). Except for cheese freshly prepared (day 0), the addition of extract increased the viability of *Lactococcus* subsp. in vacuum-packed cheeses. In contrast, the extract slightly reduced this viability for vacuum treatment combined with UV-C. Therefore, adding extract did not seem to negatively influence the lactic acid culture over the storage period of raw milk cheeses, corroborating our previous studies (25, 26).

**TABLE 1:** *Lactococcus* spp., *Enterobacteriaceae*, and *Staphylococcus* spp. counts (log cfu /g) in raw goat milk cheeses during 21 storage days at 4°C.

Bacterial groups	Treatments	Storage period (days)				
		0	6	12	18	21
*Lactococcus* subsp.	CCA	7.71 ± 0.75^Aab^	8.35 ± 0.51^Ba^	7.27 ± 0.73^Aa^	8.23 ± 0.09^Ba^	7.95 ± 0.50^Ba^
	CCV	7.09 ± 0.35^Aa^	8.20 ± 0.65^Ba^	7.65 ± 0.96^ABa^	7.28 ± 0.22^ABa^	8.07 ± 0.56^Ba^
	CCU	7.83 ± 0.20^Bb^	7.63 ± 0.19^ABa^	7.42 ± 0.12^Aa^	7.38 ± 0.75^Aa^	7.97 ± 0.40^Ba^
	CEA	7.38 ± 0.23^Aa^	8.16 ± 0.45^Ba^	8.27 ± 0.74^Ba^	7.48 ± 0.80^ABa^	8.00 ± 0.43^Ba^
	CEV	7.92 ± 0.15 ^Ab^	7.86 ± 0.62^ABa^	8.32 ± 0.41^Ba^	7.87 ± 0.19^ABa^	7.58 ± 0.58^Aa^
	CEU	7.06 ± 0.39^Aa^	7.78 ± 0.19^ABa^	7.68 ± 0.03^ABa^	7.74 ± 0.38^ABa^	8.31 ± 0.28^Ba^
*Enterobacteriaceae*	CCA	4.77 ± 0.24^Ab^	4.79 ± 0.34^Ab^	5.13 ± 0.21^Ac^	4.99 ± 0.14^Ab^	4.44 ± 0.34^Ab^
	CCV	4.38 ± 0.20 ^Bb^	3.92 ± 0.92^ABa^	1.90 ± 0.00^Aa^	1.90 ± 0.00^Aa^	1.90 ± 0.00^Aa^
	CCU	4.47 ± 0.01^Bb^	4.07 ± 0.02^Aab^	3.79 ± 0.26^Abc^	5.47 ± 0.03^Cb^	5.67 ± 0.09^Cc^
	CEA	4.52 ± 0.11^Ab^	4.31 ± 0.22^Ab^	3.03 ± 0.83^Aab^	3.31 ± 0.28^Aab^	5.60 ± 0.12^Ac^
	CEV	3.84 ± 0.09^Ca^	3.41 ± 0.34^Ba^	1.90 ± 0.00^Aa^	1.90 ± 0.00^Aa^	1.90 ± 0.00^Aa^
	CEU	4.41 ± 0.29^Ab^	4.24 ± 0.11^Ab^	5.41 ± 0.26^Bc^	5.48 ± 0.27^Cb^	5.64 ± 0.28^Cc^
*Staphylococcus* subsp.	CCA	4.75 ± 0.35^Aa^	5.59 ± 0.18^Bb^	4.77 ± 0.40^Ab^	5.95 ± 0.02^Bb^	4.28 ± 0.39^Ac^
	CCV	4.74 ± 0.51^ABa^	5.59 ± 0.05^Bb^	5.76 ± 0.87^Bbc^	5.84 ± 0.22^Bb^	3.80 ± 0.02^ABc^
	CCU	4.22 ± 0.68^Aa^	5.24 ± 0.09^Ba^	6.14 ± 0.52^Cc^	3.76 ± 0.03^Aab^	3.76 ± 0.03^Aab^
	CEA	4.73 ± 0.39^ABa^	5.34 ± 0.11^Cab^	4.76 ± 0.12^ABb^	5.24 ± 0.14^BCb^	4.41 ± 0.05^Ac^
	CEV	4.92 ± 0.54^Ba^	5.47 ± 0.03^Bab^	1.90 ± 0.00^Aa^	1.90 ± 0.00^Aa^	1.90 ± 0.00^Aa^
	CEU	4.91 ± 0.37^Aa^	5.38 ± 0.01^Aab^	5.37 ± 0.21^ABc^	5.07 ± 0.69^Ab^	4.49 ± 0.26^Ac^

*CCA = raw milk cheese without extract in aerobic packaging, CCV = raw milk cheese without extract in vacuum packaging, CCU = raw milk cheese without extract in vacuum packaging + UV-C, CEA = raw milk cheese with extract in aerobic packaging, CEV = raw milk cheese with extract in vacuum packaging; CEU = raw milk cheese with extract in vacuum packaging + UV-C.*

*^A–C^Different uppercase superscripts indicate significant differences among storage times (P < 0.05).*

*^a–d^Different lowercase superscripts indicate significant differences among treatments (P < 0.05).*

Consistently, in CEA and CEU, there was an increase in *Lactococcus* in 6 and 21 days, respectively, with both treatments ending storage with counts up to 1.18-fold higher than fresh cheese. For CEV, subsequently to the rise in *Lactococcus* in 12 days, there was a reduction in 21 days of storage. However, the value at the end of storage was equal to fresh cheese. Similar behavior was found for treatments without adding extract, where CCA and CCV ended the hold with *Lactococcus* counts up to 1.14-fold higher than fresh cheese. In contrast, CCU ended the period with a similar count. Coherently, there was an interaction between the storage period and preservation method for *Lactococcus* count (*P* = 2.8 × 10^–4^). Inline, Dupas et al. (37) demonstrated that natural extracts did not affect the starter culture growth in fresh goat cheese. Although pequi has antimicrobial action, extract molecules from it act specifically on pathogenic bacteria, such as proteins responsible for virulence factors or enzymes not found in LAB (38). Still, Murtaza et al. (39) indicated that decreasing NaCl content could favor the lactic acid bacteria’s growth. Finally, cheeses with or without extract presented counts greater than 7 log cfu/g, regardless of treatment, meeting viability requirements (40) throughout storage.

Regarding *Enterobacteriaceae*, all cheeses presented initial counts ranging between 3.84 ± 0.09 and 4.77 ± 0.24 log cfu/g. These counts were expected, and are related to the non-pasteurization of milk for cheese production, since one of the objectives of the study is to evaluate the conservation methods in the microbiota from raw milk. The vacuum was the most effective treatment against *Enterobacteriaceae*, with and without pequi extract, when compared to other preservation methods. Based on this, Siripatrawan and Harte (41) related that vacuum could increase intermolecular interaction between antimicrobial compounds naturally present in the matrix and microorganisms. It may have contributed to the slowing microbial growth of *Enterobacteriaceae*. However, the vacuum treatment started to show effectiveness for cheese without extract only from the 6th day of storage. On the other hand, the extract enabled the reduction of *Enterobacteriaceae* by vacuum, even in fresh cheese. On the 6th day of storage, the extract remained to show an effect with the vacuum, so that CEV had an *Enterobacteriaceae* count up to 1.26-fold lower than CEA and CEU. In contrast, although CCV has been lower 1.22-fold than CCA for cheeses without extract, the enterobacteria’s former count was similar to CCU. These findings are consistent with interaction between preservation method and storage found for *Enterobacteriaceae* growth herein (*P* = 1.90 × 10^–13^). The intermolecular interaction of antimicrobial compounds from pequi and microorganisms has been probably accelerated by vacuum, thereby reducing *Enterobacteriaceae*’s growth since the beginning of storage.

However, the extract seemed to start to lose its antibacterial activity from 12 days of cheese storage. Although CEV has remained lower than CEU (2.85-fold) during this storage time, its count became similar to CEA. This behavior stayed for 18 days. On the other hand, for cheeses without extract, CCV was up to 2.98-fold more effective than the other treatments (CCA and CCU) from the 12th day until the end of storage. These findings suggest that bioactive compounds against *Enterobacteriaceae* in the extract from pequi start to lose their stability in the matrix from 12 days of storage. A possible strategy to increase the stability of these compounds would be to use lyophilized extract. The loss of extract stability can be seen in Supplementary Figure 1. The straight 1/slope ratio is more remarkable in CEV, reporting that the drop in enterococci counts is less accentuated for cheeses with extract than those without during 21 storage days. This is consistent with the lack of synergism (*P* = 0.458) between extract and vacuum on the growth of *Enterobacteriaceae* during cheese storage.

The antimicrobial activity of pequi extract was previously attributed to terpenes and polyphenol compounds, such as flavonoids, present in high concentrations in this fruit (24). The flavonoids activity has been reported to the bacteriostatic and bactericidal effects (42). The antimicrobial mechanism of terpenes was associated with damaging cell membrane integrity, which can occur by interactions with phospholipids releasing nucleic acids (43) or inhibition of the mitochondrial respiratory chain (44), resulting in cell death. Besides, phenols, alcohols, ketones, and ethers may interact with *Enterobacteriaceae* family members through hydrogen bonds with hydroxyl groups and receptor groups of proteins (e.g., NH and CO) in cheese. These results also could be related to antimicrobial substances (e.g., nisin, enterocins, bioactive peptides, and exopolysaccharides) produced by *Lactococcus* subsp. that could have acted synergistically with terpenes (44).

On the other hand, aerobic treatments (CCA and CEA) showed no changes during storage (*P* > 0.05). In contrast, UV-C treatments ended hold with enterobacterial counts up to 1.28 times higher than fresh cheese; for CEU, this increase was linear (Supplementary Figure 1), while for CCU, there was an increase in 18 days, preceded by a reduction in 6 days. It suggests that UV-C has a limited effect on the *Enterobacteriaceae* family. This behavior could be related to the low penetration power of UV-C radiation (45). Since *Enterobacteriaceae* come from raw milk, these microorganisms also are present in the inner of cheese. Thus, the UV-C method’s action was not enough to control the growth of this bacterial family in this foodstuff.

*Staphylococcus* subsp. also exhibited an initial value (between 4.22 ± 0.26 and 4.91 ± 0.18 log cfu/g) in all treatments. For cheeses without extract, the vacuum treatment was ineffective in reducing staphylococcal counts throughout the storage, being CCV always similar to CCA. On the other hand, UV-C treatment showed significant differences at some storage points; at 6 and 21 days, it slightly reduced staphylococcal counts compared to CCA, while at 12 days, the treatment caused a slight increase. However, CCU can continue to present a risk of toxin production (3.76 ± 0.03 log cfu/g).

In contrast, the presence of pequi extract acted synergistically with the vacuum treatment during storage (*P* = 0.018 × 10^–18^). It led to reductions of up to 2.87-fold in staphylococcal count compared to other treatments (CEA and CEU) from 12 days of product storage; none was found above the method threshold until the assay’s end. Indeed, comparing CEV and its control treatment (CCV), we observed a synergistic effect between pequi extract and vacuum from 12 storage days, so the extract resulted in up to a 3-fold reduction in staphylococcal viability. Still, pequi extract combined with vacuum was the only treatment to reduce the staphylococcal count (*P* < 0.05) compared to fresh cheese during storage. The other treatments, despite slight fluctuations, ended the hold with *Staphylococcus* subsp. viability similar to fresh cheese, while CEU had no significant changes throughout the storage.

These results corroborated the effect between the combined treatment of pequi extract and vacuum observed for *Enterobacteriaceae* and highlighted this treatment’s positive effect against potentially pathogenic microorganisms. Regarding the combined treatment with UV-C radiation, the cheese with extract had staphylococcal values equal to or 1.19-fold greater than the one without the addition of extract. These results indicate a possible degradation of the extract by radiation.

The reduction of *Staphylococcus* subsp. is interesting because it can produce staphylococcal enterotoxins. They cause food poisoning, mostly in cheeses made from raw milk (46). Moreover, the secretion of proteases and lipases by psychotropic bacteria, such as *Pseudomonas* spp., *Staphylococcus* spp. and *Enterobacteriaceae*, causing cheese quality impairment, as spoilage, shelf life reduced, undesirable effects in sensory attributes, and economic losses to the dairy industry (47), which reduces the consumer acceptance. Bukvicki et al. (48) also reported antibacterial effects against Gram-negative and Gram-positive bacterias in a soft cheese after 30 days of storage at 4°C by using thyme oil (*Thymus algeriensis*), which is rich in terpenes such as the pequi fruit.

The findings from multivariate analysis (Supplementary Figure 2) corroborated the previous results herein. The principal component 1 (PC1) corresponded to 40.84% of the data variance. It showed that, regardless of the treatment, the greater variability of the data was due to the storage time. The right quadrant concentrated samples in the initial storage period (0 and 6 days), while the left quadrant concentrated samples from 12 days of storage. Thus, *Lactococcus* values were higher in samples stored for a longer time than in fresher samples (right quadrant), corroborating the growth of *Lactococcus* in almost all treatments throughout storage.

The PC2 had a variance of 14.49%. Its lower quadrant concentrated vacuum-treated samples. In addition, this quadrant presented lower loadings for staphylococci and enterobacteria, especially in treatments with the addition of extract after 12 days. Therefore, these findings reinforced the synergistic antimicrobial effect between pequi extract and vacuum. Consistently, in the PC3 (10.79%), there was a grouping in the upper quadrant of samples with extract associated with another preservation treatments (CEV and CEU) characterized by lower loadings of enterobacteria.

### Chemical Parameters of Cheeses

Table 2 shows the proximate composition (%) of fresh cheeses (storage day 0). The cheeses without adding extract showed no difference (*P* > 0.05) between the preservation treatments regarding moisture and protein. However, the addition of extract influenced the preservation treatments, leading to different behaviors between them. All cheeses’ presented moisture values below 55% (v/w), stipulated in Brazil for Minas Frescal cheese (49). It can be due to the partial replacement of NaCl by KCl since there is a higher water-holding capacity by the protein matrix inherent to NaCl (50).

**TABLE 2:** Proximate composition (%) of raw goat milk cheeses on day 0 at 4°C.

Treatments	Moisture	Protein	Fat	Ash
CCA	53.90 ± 0.33^cd^	17.60 ± 0.67^bc^	16.40 ± 0.10^ab^	3.71 ± 0.20^b^
CCV	53.70 ± 0.32^bc^	18.36 ± 0.72^c^	18.85 ± 0.35^b^	3.30 ± 0.05^ab^
CCU	53.88 ± 1.45^cd^	18.24 ± 0.97^c^	16.65 ± 0.55^ab^	3.26 ± 0.23^a^
CEA	54.78 ± 0.82^d^	17.78 ± 0.55^bc^	15.80 ± 0.50^a^	3.56 ± 0.15^b^
CEV	52.79 ± 0.21^b^	17.79 ± 0.89^bc^	16.55 ± 0.75^ab^	3.53 ± 0.11^b^
CEU	51.80 ± 0.27^a^	16.68 ± 0.97^a^	16.35 ± 0.15^ab^	3.86 ± 0.36^c^

*CCA = raw milk cheese without extract in aerobic packaging, CCV = raw milk cheese without extract in vacuum packaging, CCU = raw milk cheese without extract in vacuum packaging + UV-C, CEA = raw milk cheese with extract in aerobic packaging, CEV = raw milk cheese with extract in vacuum packaging; CEU = raw milk cheese with extract in vacuum packaging + UV-C.*

*^a–d^Different lowercase superscripts indicate significant differences among treatments (P < 0.05).*

For cheeses with extract, treatment with UV-C combined with vacuum showed the lowest moisture. In contrast, UV-C radiation may have facilitated the salt absorption in cheeses with extract, causing a more substantial syneresis and directly influencing a greater water loss by this treatment (51). Consistently, CEU had lower 4% moisture content than the CCU control.

For protein content and considering cheeses with extract, UV-C radiation led to contents of this nutrient up to lower 7%. Inline, CEU had protein content 8.5% lower than CCU control. In contrast, CEU was the treatment with the highest ash content among cheeses with added extract – up to 9% – with no difference between the other treatments (*P* > 0.05). Inline, ash content in CEU was 1.18 times higher compared to CCU. These findings are consistent with the inverse correlation found here between protein and ash values – *R* = −0.943; *P* = 0.005 – (Supplementary Figure 1). As CEU had higher moisture loss (Table 2), it can be inferred that it suffered a greater protein loss through whey, which is consistent with the fact that salt is the main component of ash, and the higher the salt content, the greater the loss of moisture (51). In contrast, UV-C treatment had the opposite effect on non-extract cheeses, such that CCU had 1.14 times less ash content than CCA. This opposite behavior corroborates the extract’s influence in modifying the cheese’s chemical composition through different preservation methods.

Regarding the fat content, there were no significant changes (*P* > 0.05) within the groups with and without extract, nor between them when comparing similar preservation methods (Table 2). Unlike proteins, fat is not soluble in water, which helps prevent it from being lost through whey.

**FIGURE 1:**
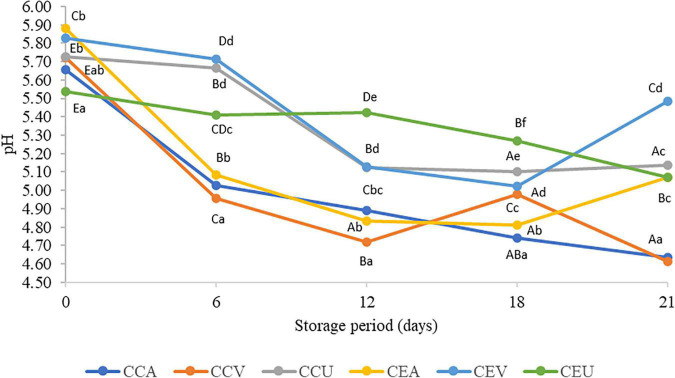
Values of titratable acidity in raw goat milk cheeses during 21 days of storage at 4°C. CCA = raw milk cheese without extract in aerobic packaging, CCV = raw milk cheese without extract in vacuum packaging, CCU = raw milk cheese without extract in vacuum packaging + UV-C, CEA = raw milk cheese with extract in aerobic packaging, CEV = raw milk cheese with extract in vacuum packaging; CEU = raw milk cheese with extract in vacuum packaging + UV-C. A–D Different lowercase superscripts indicate significant differences among storage times (*P* < 0.05). a–d Different uppercase superscripts indicate significant differences among goat milk cheeses (*P* < 0.05).

In general, the treatments ended the storage more acidic than the fresh cheese (day 0), regardless of the presence or absence of extract. The CEV treatment exception ended hold with a pH similar to fresh cheese (Figure 1). Post-acidification was corroborated by PC1, in which the samples, regardless of treatment, had higher pH scores at the beginning of storage – 0 and 6 days – (Supplementary Figure 2). The reduction in pH is due to post-acidification during storage, linked to the progressive transformation of lactose into lactic acid by LAB (52). However, the extract delayed the post-acidification of cheeses for vacuum and aerobic treatments. Thus, CEV had a pH value up to 19 % higher than CCV from 6 days of storage, while CEA was up to 9% less acidic than CCA.

In contrast, in general, UV-C radiation combined with vacuum did not affect the cheese’s pH value (*P* > 0.05) compared to the control without extract. A notable difference in behavior during storage was the increase in pH from 18 to 21 days for the treatments with extract (CEA and CEV), while CCA remained stable and CCV showed a sharp drop. On the other hand, CEU maintained a stable pH from 18 to 21 days and its respective CCU control. More similar behavior can justify similar pH values for CCU and CEU throughout almost all storage. Inline, the treatments interacted during storage (*P* = 7.4 x 10^–68^). Consistently, PC3 had higher pH scores in the upper quadrant, which grouped samples with extract (Supplementary Figure 2).

Post-acidification kinetics depend on milk composition, total solids level, and interaction among milk constituents (53). In this context, it is essential to note that the extract led to significant differences in the chemical composition of cheeses, which may have contributed to delay post-acidification in these products (Table 2). This delayed post-acidification can improve cheese quality since acidification during storage depreciates the quality of dairy products, reducing their shelf life, decreasing consumer acceptance, and even detrimental to the stability of probiotics (53).

Still, for cheeses with extract, aerobic treatment was the main responsible for post-acidification during almost all storage (6–18 days). Thus, CEA was up to 1.12-fold more acidic than the other treatments (CEV and CEU). The availability of oxygen in aerobic packaging can promote acidity due to increased lipid oxidation and consequent release of acidic compounds favored by oxygen presence (45). In contrast, vacuum treatment was as effective for cheeses without extract as aerobic treatment in post-acidifying the cheese from 6 to 21 days. Vacuum-packaged cheeses can accumulate lactic, formic, acetic, and butyric acid compared with non-vacuum-packaged cheeses (54), possibly due to lipids hydrolysis increased during storage (55). Thus, the addition of pequi extract seems to reduce post-acidification favored by vacuum. Inline, PC1 shows a decrease in pH scores along storage period, especially for samples with added extract and vacuum packed (Supplementary Figure 2).

**FIGURE 2:**
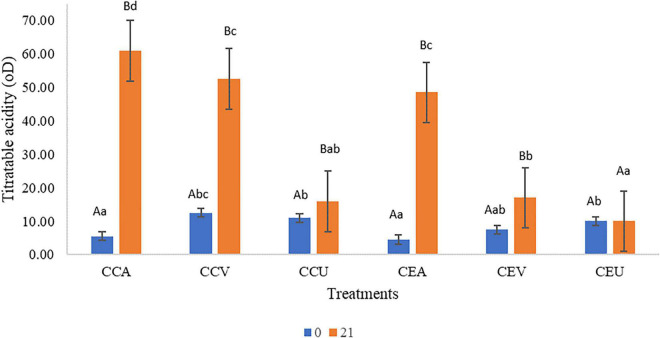
Raw goat milk cheeses’ pH values during 21 storage days at 4°C. CCA = raw milk cheese without extract in aerobic packaging, CCV = raw milk cheese without extract in vacuum packaging, CCU = raw milk cheese without extract in vacuum packaging + UV-C, CEA = raw milk cheese with extract in aerobic packaging, CEV = raw milk cheese with extract in vacuum packaging; CEU = raw milk cheese with extract in vacuum packaging + UV-C. A–D Different lowercase superscripts indicate significant differences among storage times (*P* < 0.05). a–d Different uppercase superscripts indicate significant differences among goat milk cheeses (*P* < 0.05).

Titratable acidity findings (Figure 2) corroborated the results obtained for pH (Figure 1), there is interaction between the preservation methods and time (*P* = 1.2 × 10^–20^). Regardless of the addition of extract, the treatments had increased up to 12-fold titratable acidity throughout storage. CEU was an exception since it remained similar to fresh cheese (day 0). However, post-acidification was more intense for cheeses without extract; CCA had acidity higher 1.2-fold than CEA at the end of storage, while CCV showed a higher 2.68-fold value than CEV. The exception was for UV-C treatment, where CCU was similar to CEU. Still consistent with the results found for pH (Figure 1), aerobic packaging was the primary treatment responsible for acidification, followed by vacuum regardless of the addition of extract. The least effective method in this parameter was the UV-C (Figure 2).

### Instrumental Color Characteristics

**FIGURE 3:**
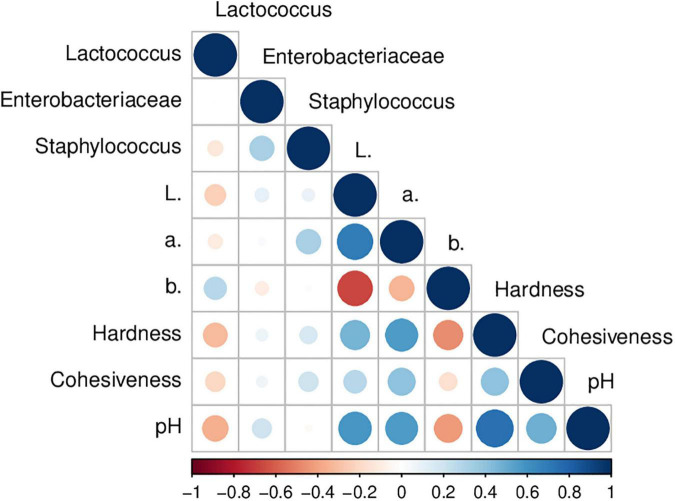
Pearson’s correlation coefficients among physicochemical and microbial quality parameters of raw goat milk cheeses stored at 4°C. Correlations with *P*-value > 0.05 are considered insignificant.

Table 3 presents the color results of goat Minas Frescal cheese during storage. Color is an essential parameter to be evaluated in food products because of its direct effect on appearance and consumer acceptance (32). The L* value, which measures whiteness, results from colloidal particles in milk, including fat globules and casein micelles, which can scatter light in the visible spectrum (56). In general, there was a reduction up to 6% in L* values at the end of storage compared to fresh cheese; this reduction was linear (*P* < 0.05) for CCA, CCV, CEA, and CEU (Supplementary Figure 1). For CCU, the non-linear reduction behavior may be due to this treatment presenting an increase in L* in 12 days, which preceded the reduction in 18 days. Inline, regardless of treatment, lower L* scores were obtained in PC1 for samples at the end of storage (Supplementary Figure 2). A decrease in the light scattering degree (L* values) during storage was previously reported for cheese (40) and also for cheese incorporated with plant extracts containing flavonoids (57). It can be attributed to post-acidification (Figure 1). Values of pH and L* showed a positive correlation (*R* = 0.60; *P* = 5 × 10^–4^) herein (Figure 3). Acidic pH decreases the net charge of caseins and causes the solubilization of colloidal calcium phosphate from the micelles into the solution. Consequently, there is a shrinkage of the micelles and their dissociation (58), reducing the cheese’s ability to scatter light (56).

**TABLE 3:** Instrumental color and texture characterization of raw goat milk cheeses during 21 days of storage at 4°C.

Parameters	Treatments	Storage period (days)				
		0	6	12	18	21
L*	CCA	93.15 ± 0.04^BCa^	92.55 ± 0.76^Bbc^	92.05 ± 0.89^ABc^	89.10 ± 0.56^Ab^	88.56 ± 0.44^Ab^
	CCV	93.99 ± 0.72^Ca^	92.81 ± 0.24^BCbc^	90.28 ± 1.47^ABab^	88.71 ± 1.44^Aab^	89.43 ± 0.01^Aabc^
	CCU	94.23 ± 0.41^BCa^	93.26 ± 0.34^ABc^	95.09 ± 0.39^Ce^	92.38 ± 0.55^Ac^	92.34 ± 0.39^Ad^
	CEA	92.78 ± 0.88^Ba^	92.58 ± 0.23^Bbc^	89.34 ± 0.42^Aa^	87.37 ± 0.62^Aab^	87.18 ± 2.05^Aa^
	CEV	92.88 ± 1.20^Ba^	92.70 ± 0.28^Bbc^	93.05 ± 1.00^Bd^	87.18 ± 1.58^Aa^	92.13 ± 1.12^Bd^
	CEU	92.62 ± 0.60^Ba^	89.81 ± 1.15^ABa^	91.02 ± 0.59^Abb^	88.74 ± 1.02^ABab^	88.17 ± 0.62^Aab^
a*	CCA	1.23 ± 0.13^Ba^	1.18 ± 0.04^Ba^	1.19 ± 0.04^Ba^	−1.21 ± 0.08^Abc^	−1.58 ± 0.05^Aa^
	CCV	1.19 ± 0.12^Ba^	1.47 ± 0.09^Bb^	1.28 ± 0.08^Bb^	−1.39 ± 0.11^Aa^	−1.54 ± 0.08^Aab^
	CCU	1.45 ± 0.08^Bb^	1.84 ± 0.05^Dd^	1.49 ± 0.12^BCc^	−1.08 ± 0.12^Acd^	−1.12 ± 0.17^Ac^
	CEA	1.88 ± 0.05^Dd^	1.80 ± 0.05^CDcd^	1.38 ± 0.07^Bc^	−1.32 ± 0.16^Aab^	−1.32 ± 0.05^Ab^
	CEV	1.77 ± 0.28^BCcd^	2.02 ± 0.07^BCe^	1.54 ± 0.12^Bc^	−1.31 ± 0.03^Aab^	−0.76 ± 0.27^Ad^
	CEU	1.99 ± 0.03^Bd^	2.12 ± 0.09^Be^	1.82 ± 0.05^Bd^	−0.81 ± 0.26^Ad^	−1.14 ± 0.16^Ac^
b*	CCA	8.25 ± 0.58^Aa^	8.98 ± 0.23^Aa^	9.56 ± 0.79^ABa^	10.34 ± 0.18^Bab^	10.02 ± 0.38^Bab^
	CCV	8.29 ± 0.70^Aa^	9.49 ± 0.27^ABa^	10.24 ± 1.27^Ba^	9.16 ± 0.90^Aa^	9.99 ± 0.76^Bab^
	CCU	50 ± 0.29^Aa^	10.76 ± 0.32^Ba^	8.78 ± 0.34^Aa^	9.38 ± 0.23^ABa^	9.03 ± 0.98^Aba^
	CEA	8.30 ± 0.71^Aa^	10.02 ± 0.35^BCa^	9.92 ± 0.38^BCa^	10.40 ± 0.53^Ca^	10.60 ± 0.21*^Cabc^*
	CEV	8.66 ± 0.59^Aa^	10.12 ± 0.78^Ba^	9.33 ± 0.95^Aa^	10.26 ± 1.16^Ba^	10.29 ± 0.95^Babc^
	CEU	9.04 ± 0.47 ^Aa^	10.79 ± 0.03^Ba^	10.00 ± 0.26^Ba^	10.93 ± 0.81^Bab^	10.14 ± 0.64^BaBc^
ΔE	CCA	NA	1.09 ± 0.21 ^Aa^	1.97 ± 0.36 ^Aac^	5.61 ± 0.62 ^Ba^	5.57 ± 0.13 ^Ba^
	CCV	NA	2.10 ± 0.07 ^Aa^	4.68 ± 0.59 ^Bb^	7.13 ± 1.26 ^Ca^	6.06 ± 0.07 ^BCab^
	CCU	NA	2.96 ± 0.004 ^ABa^	1.32 ± 0.28 ^Aa^	3.03 ± 0.32 ^ABb^	3.70 ± 0.27 ^Bc^
	CEA	NA	2.34 ± 0.19 ^Aa^	3.46 ± 0.63 ^Abc^	6.64 ± 0.003 ^Ca^	7.77 ± 0.45 ^Cb^
	CEV	NA	1.12 ± 0.69 ^Aa^	1.04 ± 0.30 ^Aa^	5.61 ± 0.87 ^Ba^	2.84 ± 0.25 ^Ac^
	CEU	NA	1.83 ± 0.61 ^Aa^	1.27 ± 0.58 ^Aa^	5.61 ± 0.11 ^Ba^	5.17 ± 0.49 ^Bad^
ΔC*	CCA	NA	0.637 ± 0.112 ^Aa^	1.25 ± 0.06 ^Aa^	1.65 ± 0.14 ^Aab^	1.68 ± 0.18 ^Aab^
	CCV	NA	1.51 ± 0.02 ^Aac^	2.35 ± 0.08 ^Bb^	1.75 ± 0.68 ^Aab^	1.21 ± 0.50 *^Aac^*
	CCU	NA	2.71 ± 0.04 ^Bb^	0.54 ± 3x10^–3 Aac^	0.938 ± 0.198 ^Aa^	1.30 ± 0.26 ^Aac^
	CEA	NA	2.09 ± 0.32 ^Abc^	2.11 ± 0.03 ^Ab^	1.55 ± 0.55 ^Aab^	2.48 ± 0.37 ^Ab^
	CEV	NA	2.16 ± 0.90 ^Bbc^	1.25 ± 0.68 ^ABac^	2.23 ± 0.37 ^Bb^	0.647 ± 0.402 ^Ac^
	CEU	NA	1.99 ± 0.20 ^Abc^	1.20 ± 0.05 ^Aac^	1.92 ± 0.29 ^Ab^	1.05 ± 0.11 ^Aac^
Hardness (*g*)	CCA	507.07 ± 22.71^Da^	327.56 ± 22.86^Cbc^	96.16 ± 6.86^Aa^	130.63 ± 9.13^ABb^	164.06 ± 24.57^Bcd^
	CCV	601.48 ± 33.65^Db^	219.30 ± 21.70^BCa^	247.03 ± 6.83^BCd^	173.10 ± 13.74^ABab^	121.12 ± 10.10^Abc^
	CCU	499.70 ± 21.85^Da^	509.19 ± 34.73^Dd^	186.33 ± 14.93^BCc^	211.60 ± 56.28*^Cbc^*	63.02 ± 4.57^Aa^
	CEA	501.39 ± 33.44^Ea^	221.23 ± 17.06^BCa^	337.98 ± 65.50^De^	95.19 ± 5.68^Aa^	159.13 ± 9.21^ABc^
	CEV	793.26 ± 43.95^Bcd^	358.04 ± 52.19^Ac^	132.13 ± 13.64^Aab^	226.00 ± 24.60^Acd^	173.61 ± 14.04^Acd^
	CEU	706.34 ± 45.18^Cc^	296.51 ± 18.20^Bbc^	147.56 ± 11.36^Abc^	335.40 ± 41.27^Bde^	112.44 ± 8.08^Aab^
Cohesiveness (*g*)	CCA	1.49 ± 0.04^Cb^	1.29 ± 0.11^BCab^	1.10 ± 0.14^ABa^	1.04 ± 0.02^Aa^	1.00 ± 0.05^Aab^
	CCV	1.31 ± 0.11^Bb^	1.27 ± 0.11^Bab^	1.01 ± 0.14^Aa^	1.32 ± 0.19^Bab^	1.11 ± 0.09^Bab^
	CCU	1.39 ± 0.08^Ab^	1.37 ± 0.03^Aabc^	1.32 ± 0.15^Aa^	1.02 ± 0.12^Aa^	1.15 ± 0.11^Aab^
	CEA	1.29 ± 0.04^Ab^	1.40 ± 0.04^Aabc^	1.20 ± 0.20^Aa^	1.30 ± 0.06^Aab^	1.10 ± 0.08^Aab^
	CEV	1.13 ± 0.28^Aab^	1.26 ± 0.28^Aab^	1.24 ± 0.24^Aa^	1.27 ± 0.21^Aab^	1.12 ± 0.06^Aab^
	CEU	1.36 ± 0.09^ABb^	1.32 ± 0.04^ABabc^	1.29 ± 0.14^Aba^	1.60 ± 0.28^Bb^	1.03 ± 0.10^Aab^

Lightness value (L*), Redness value (a*), Yellowness value (b*), Difference for color and lightness (ΔE), Difference for color’s saturation (ΔC*). NA = not applied; CCA = raw milk cheese without extract in aerobic packaging, CCV = raw milk cheese without extract in vacuum packaging, CCU = raw milk cheese without extract in vacuum packaging + UV-C, CEA = raw milk cheese with extract in aerobic packaging, CEV = raw milk cheese with extract in vacuum packaging; CEU = raw milk cheese with extract in vacuum packaging + UV-C.

*^A–C^Different uppercase superscripts indicate significant differences among storage times (P < 0.05).*

*^a–d^Different lowercase superscripts indicate significant differences among treatments (P < 0.05).*

In contrast, cheese with extract combined with vacuum ended storage with L* value similar to fresh cheese. Thus, CEV was the most efficient treatment in maintaining whiteness during storage. Indeed, for cheeses with extract, L* values for CEV were up to 6 % higher than CEA and CEU during storage (Table 1). The potential of this treatment to delay post-acidification may justify this finding since it was the only one capable of finishing the storage with pH similar to that of fresh cheese (Figure 1). Consistently, L* values for CEV were identical or up to higher 1.19-fold than CCV control during storage. These findings were corroborated by the multivariate analysis, in which cheeses with extract and vacuum-packed, especially from 12 days, had the highest L* values (negative scores in PC2, see Supplementary Figure 2). The antioxidant activity (24) and the inhibition of lipases by carotenoids of pequi extract (59) may have contributed to mitigate the post acidification by different preservation methods compared to cheeses without extract, also influencing the color parameters (60).

On the other hand, for cheeses without adding extract, CCU presented L* values up to a higher 5% compared to CCA and CCV treatments from 12 days. It can be attributed to UV-C radiation combined with the vacuum being the most effective treatment in delaying post-acidification for cheeses without extract. The ability of the combination with UV-C to delay post-acidification by vacuum during storage has been previously reported in fish meat (61). Therefore, CCU was less acidic than CCA and CCV from 6 days of storage (Figure 1). Coherently, CCU had L* values up to higher 5% compared to CEU (Table 3), confirmed by negative scores for CCU in PC3 and positive scores for CEU (Supplementary Figure 2). In contrast, aerobic packaging resulted in a more accentuated loss of lightness values, which may be related to increased acidification during storage compared to other preservation methods, which directly contributed to the reduction of its whiteness (Figure 3).

Despite fluctuations during storage, the period ended with a* reduction in all treatments (Table 3); this decrease was up to 1.29-fold compared to fresh cheese. For CEA (*R* = −0.908; *P* = 0.03) and CEU (*R* = −884; *P* = 0.04) this reduction was linear with time (Supplementary Figure 1). Indeed, samples at the beginning of storage – 0 and 6 days – had higher scores of a* than other days in PC1 (Supplementary Figure 2). This behavior was associated with post-acidification of the cheeses, as a direct correlation – *R* = 0.562; *P* = 0.001 – was observed between pH and a* values herein (Figure 3). It is well established that the color of fermented dairy products also depends on pH since the acidification of the cheese leads to a greater syneresis. It can induce a decrease in a* values because the whey released by the gel contains riboflavin, which has a very important green component (56). A decrease in a* values with storage and its positive correlation with pH was previously reported for goat’s fermented milk (62) and also for goat’s cheese added with essential oil (63).

Inline, among the treatments with extract, CEU and CEV were those that presented higher pH values during storage (Figure 1) and, consequently, the importance of a* was up to higher 1.70-fold. The highest a* scores were found for CEU after 6 days in PC2 (Supplementary Figure 2). Accordingly, CCU, the treatment with the most elevated pH among cheeses without extract, was also the sample with the highest a*, reaching a value up to higher 1.64-fold than the others. Furthermore, as adding extract delayed post-acidification, these cheeses had a* values up to higher 1.53-fold than their respective controls without extract. Indeed, samples with extract had higher scores for a* in PC3 (Supplementary Figure 2). Finally, as a* and L* correlated directly with the pH values, these two parameters of color were also associated with each other – *R* = 0.691; *P* < 0.0001 – Figure 3.

In general, cheeses had increased b* chroma up to 1.28-fold, regardless of the presence or absence of pequi extract. The exception was the CCU treatment, which ended the period with a b* value similar to that of fresh cheese (Table 3). As illustrated in Figure 3, the b* value had a negative correlation with pH (*R* = −0.425; *P* = 0.02) and L* (*R* = −0.661; *P* < 0.0001). Thus, post-acidification and darkening of the samples during storage contributed to an increase in b*. Indeed, cheeses stored for 12 days, regardless of the treatment, had higher b* scores in PC1 (Supplementary Figure 2). Changes of pH have an important effect on pigments naturally present in food, besides to affect some physicochemical parameters that can lead to an impaired b* during storage (64).

For cheeses without extract, CCU obtained b* values up to lower 1.11-fold in 18 and 21 days, consistent with this treatment presenting higher pH and L* values than CCA and CCV during storage. On the other hand, among cheeses with the addition of extract, CEV gave a b* value lower 6% in 18 days, which is consistent with this treatment being one of the most efficient in delaying post-acidification with the highest L* value. Inline, CEV and CEU had the lowest and highest b* scores, respectively, in PC2 (Supplementary Figure 2). In addition, cheeses with extract had b* values up to 1.16-fold higher than their respective controls without extract at the end of storage (18 and 21 days). This behavior can be attributed to the pequi extract containing carotenoids, leading to a more yellow product (24). Thus, cheeses with extract, especially from 18 days, had higher scores for yellowness than those without extract in PC3 (Supplementary Figure 2). This tendency to yellowish color is in agreement with that previously reported for goat’s cheese added with rosemary essential oil, which also contain carotenoids (63). Finally, the interaction among treatments and storage time had a greater significance for L* and a* color parameters (*P* = 3 × 10^–8^ and 1.7 × 10^–5^, respectively), and to a lesser extent, for the parameter b* (*P* = 0.046).

Table 3 shows the ΔE values during storage. In general, the treatments ended hold with increased values of ΔE up to 5.11-fold. Still, this increase was linear with time for the aerobic treatments (CCA and CEA) (Supplementary Figure 1). In contrast, CCU and CEV, despite suffering fluctuations in these values, were the only treatments able to finish storage with ΔE similar to that of the 6th day. It indicates that cheeses treated with CCU and CEV were the most stable for color. However, it is essential to highlight that the color change was visible to consumers – ΔE > 3 – for all treatments (33).

CCU had up to 3.54-fold more color stability for cheeses without extract than CCV, which was the most altered treatment. However, in the presence of extract, CEV presented up to 3.32-fold more stability than CEA, which was those with more alteration. Vacuum leads to the accumulation of organic acids (54), which has been attributed to the lipid hydrolysis resulting from this preservation method (55). It results in post acidification and color change (Figure 3). However, the addition of extract could increase the color stability in the vacuum treatment probably due to the inhibition of lipases by carotenoids, one of the major compounds present in pequi extract (59). Consequently, when comparing cheeses with added extract with those without, CEV had up to 4.5-fold greater color stability than CCV, while CCU had colored up to 1.85-fold more stable than CEU (Table 3). It demonstrates that the pequi extract influenced the color stability by combining it with the other preservation methods tested herein. Inline, the different preservation methods interacted with storage for ΔE (*P* < 0.0001). As previously discussed, CCU and CEV were the treatments with greater stability for the color parameters L*, a*, and b* during storage, which directly influenced the greater color stability of these treatments compared to the others. Indeed, the ΔE variable correlated directly with changes in L* (*R* = 0.915; *P* < 0.0001), a* (*R* = 0.835; *P* < 0.0001), and to a lesser extent, with changes in b* (*R* = 0.456; *P* = 0.025) – Supplementary Figure 1). These results corroborate with Ricciardi et al. (65), who reported that applying light technologies in combination with proper refrigeration and packaging conditions could contribute to cheese’s color preservation.

Values of chroma variation (ΔC*) are presented in Table 3. In general, these values remained constant or, in the case of CCV, despite a slight increase in 12 days, the subsequent days had a variation of C* similar to cheese at 6 days. Thus, color saturation variations tended to remain constant for almost all treatments. The exceptions were the CCU and CEV treatments, which were reduced by up to 5 and 3-fold, respectively (Table 3). Therefore, these treatments promoted more excellent stability of cheese color saturation throughout storage. Consistently, these treatments were the most effective in mitigating the increase in b* chroma during storage, which positively affected the saturation stability – *R* = 0.636; *P* = 8 × 10^–4^ – (Supplementary Figure 1).

Consequently, among the non-extracted cheeses, CCU tended to have a lower variation of C* from 12 to 21 days, with a 4.3-fold value significantly lower than CCV in 12 days. In contrast, CEV had values equal to CEU and up to 3.8-fold lower than CEA for cheeses with the added extract. Coherently, CEV had a 1.9-fold more stable color saturation than CCV at 12 days, and CCU had a major stability 2.4-fold at 18 days compared to CEU. Indeed, there was interaction of treatments with storage for ΔC* (*P* < 0.0001).

### Instrumental Texture Analysis

Table 3 shows the instrumental texture parameters of goat Minas Frescal cheese. Regardless of the extract’s presence and combined treatment, the cheese’s hardness was reduced up to 7.9-fold throughout storage. For CCU, this hardness loss was linear – *R* = −0.920; *P* = 0.03 – with time (Supplementary Figure 1). Hardness had a strong and direct correlation with pH – *R* = 0.749; *P* < 0.0001 – (Figure 3). Thus, post-acidification contributed to the softening of the cheese during storage. Indeed, from 12 days onward, cheese samples had lower pH scores, see PC1, and consequently, minor hardness than cheese between 0 and 6 days (Supplementary Figure 2). In addition, according to Supplementary Figure 1, hardness presented inverse correlation with moisture (*R* = −0.816; *P* = 0.02). The calcium solubilization in acidic pH decreases protein-to-protein interactions in cheese, making it crumblier; therefore, lowering the pH of cheese alters protein interactions, which then affects its functionality (66).

Among cheeses without extract, CCV was up to 2.57-fold harder than the other treatments. Although it did not reach statistical significance, CCV tended to have greater moisture loss than CCA and CCU (Table 2), which may have contributed to the greater hardness (Supplementary Figure 1). Similarly, greater hardness has been presented for vacuum treatment in cheeses whose extract was added. CEV showed up to 3.37-fold greater hardness than the other treatments with the extract. The interaction (*P* = 1.95 × 10^–19^) between preservation methods and storage for hardness justifies the different behaviors observed among treatments. Although CEU lost more moisture than CEV (Table 2), the latter was the most effective treatment in delaying post-acidification (Figure 1), which directly contributed to the greater hardness (Figure 3). Inline, CEV, especially from 12 days, had the highest pH and hardness scores in PC2 (Supplementary Figure 2). Therefore, although pH and moisture interfere with the cheese’s hardness, one parameter can stand out from the other.

In general, CEV and CEU were harder throughout storage than their respective controls without extract; CEV was up to 1.63-fold harder than CCV, while CEU showed hardness similar or even higher 1.58-fold than CCU (Table 3). It can be attributed to the ability of pequi extract to delay the post-acidification of cheeses (Figures 1, 2). Indeed, cheeses with extract had the highest scores for hardness and pH in PC3 (Supplementary Figure 2). Finally, as illustrated in Figure 3, cheese hardness was correlated with L* (*R* = 0.466; *P* = 0.009) and a* (*R* = 0.566; *P* = 0.001) color parameters. The loss of colloidal calcium phosphate from casein submicelles and the consequent dissociation of them due to acidity is a factor that influences both hardness (66) and color parameters (58) in cheeses. Consistently, a significant correlation among pH, moisture, hardness, and color parameters has previously been reported in cheese (67).

Cohesiveness, in general, was not affected by storage time; CCU, CEA, and CEV did not show significant changes. Despite slight modifications, CCV and CEU ended the storage similarly to fresh cheese (Table 3). In contrast, as reported in Supplementary Figure 1, cohesiveness linearly reduced for CCA (*R* = −0.974; *P* = 0.005). The greater rising in acidity in CCA (Figure 2) seems to modify the cohesiveness (Table 3) since it had a direct relationship with pH – *R* = 0.482; *P* = 0.007 – (Figure 3). In addition, CCA presented high scores for cohesiveness compared to other treatments without extract and CEV in PC2 (Supplementary Figure 2).

However, there was no difference in cohesiveness for cheeses within the treatment with extract or without it for similar day by ANOVA. Furthermore, overall, there was also no difference between these groups for similar preservation methods within the same day. However, through the PCA, it was possible to observe higher cohesiveness scores in the samples at the beginning of storage (0 and 6 days), regardless of the presence of extract and the conservation method employed. Also, the PCA analysis found higher cohesiveness scores for cheeses with pequi extract (Supplementary Figure 2). It agrees with the interaction of preservation methods with storage (*P* = 4 × 10^–4^). In addition to these findings being consistent with higher pH values at the beginning of storage and extract treatments (Figure 1), they also agree with the direct correlation between cohesiveness and hardness – *R* = 0.405; *P* = 0.03 – (Figure 3).

## Conclusion

The addition of pequi waste extract led to differences in the moisture and protein content of the cheeses between the different preservation methods. Still, the extract had no antimicrobial effect by itself. In contrast, it was synergistic with vacuum on reducing *Staphylococcus* counts in raw cheese. However, despite its initial effect on Enterobacteriaceae, bioactive compounds against this bacterial group lost their activity during cheese’s storage. The antimicrobial effects were specific against pathogenic bacteria, so that the addition of extract to any combined preservation method having no detrimental impact on *Lactococcus* starter culture. In contrast, extract with UV-C radiation favored the growth of pathogenic bacteria compared to the uncombined treatment, possibly due to its degradation by radiation. Still, this combination preferred salt absorption in cheeses, resulting in consequent protein loss due to increased syneresis in CEU. Pequi delayed post-acidification in the cheeses, which mitigated the loss of their hardness and cohesiveness resulting from storage. Nevertheless, this texture preservation was more pronounced when the extract was combined with vacuum. The extract’s efficiency to delay post-acidification also contributed to preserve the lightness and redness of the cheeses during storage, so that CEV presented more dramatic stability for the cheese’s color preservation and saturation throughout the storage. It is important note, however, that the extract led to a slight yellowing of the cheeses, due to the presence of carotenoids. Together, these results suggest the pequi extract as a vacuum potentiating agent on the microbial and physicochemical preservation of raw cheeses. This is a previous study, so that further sensory research of these cheeses will be useful for future industrial application.

## Data Availability Statement

The original contributions presented in the study are included in the article/Supplementary Material, further inquiries can be directed to the corresponding author.

## Author Contributions

RM, CV, MC, CC-J, and SM: conception and design of study. RM, RL, DG, VC, and YM: acquisition of data. RM, CV, KD, DG, AR, and MC: analysis and/or interpretation of data. RM, CV, MC, AR, and RL: drafting the manuscript. CC-J, SM, VC, YM, KD, CV, and DG: revising the manuscript critically for important intellectual content. All authors contributed to the article and approved the submitted version.

## Conflict of Interest

The authors declare that the research was conducted in the absence of any commercial or financial relationships that could be construed as a potential conflict of interest.

## Publisher’s Note

All claims expressed in this article are solely those of the authors and do not necessarily represent those of their affiliated organizations, or those of the publisher, the editors and the reviewers. Any product that may be evaluated in this article, or claim that may be made by its manufacturer, is not guaranteed or endorsed by the publisher.
