# Supplementary material for: Pequi (Caryocar brasiliense) Waste Extract as a Synergistic Agent in the Microbial and Physicochemical Preservation of Low-Sodium Raw Goat Cheese
Source: Front Nutr. 2022 Apr 6;9:855115. doi: 10.3389/fnut.2022.855115 (PMC9020873; doi:10.3389/fnut.2022.855115)
Supplement: Supplementary file 1 [file Data_Sheet_1.docx]

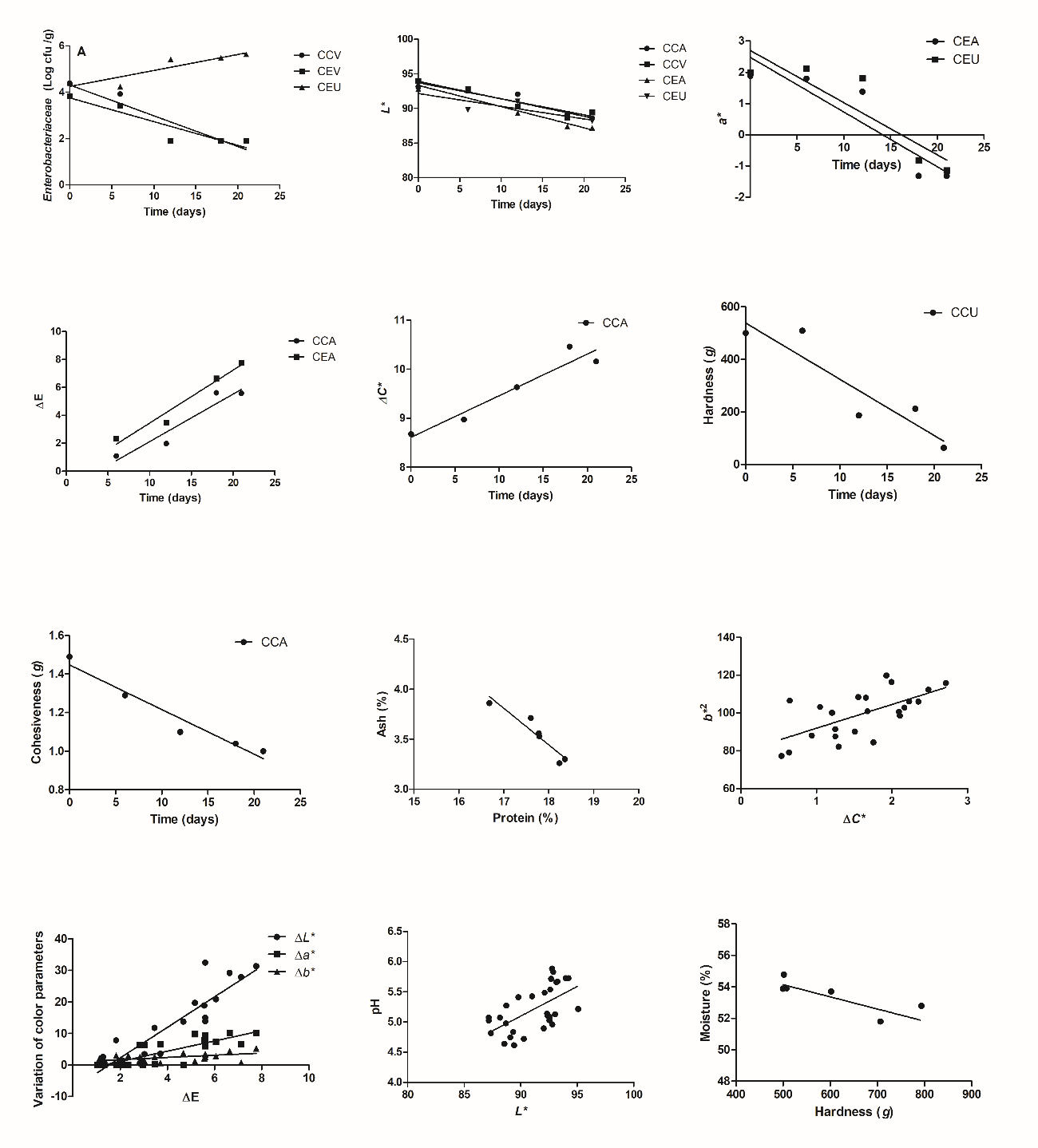


Figure S1. Significant correlations (*P* ˂ 0.05) for physicochemical and microbial quality parameters of raw goat milk cheeses during 21 storage days at 4 °C. *L**: lightness; *a**: greenness; *b**: yellowness; ΔE: difference for color and lightness; Δ*C**: difference for color´s saturation; CCA = raw milk cheese without extract in aerobic packaging, CCV = raw milk cheese without extract in vacuum packaging, CCU = raw milk cheese without extract in vacuum packaging + UV-C, CEA = raw milk cheese with extract in aerobic packaging, CEV = raw milk cheese with extract in vacuum packaging; CEU = raw milk cheese with extract in vacuum packaging + UV-C.


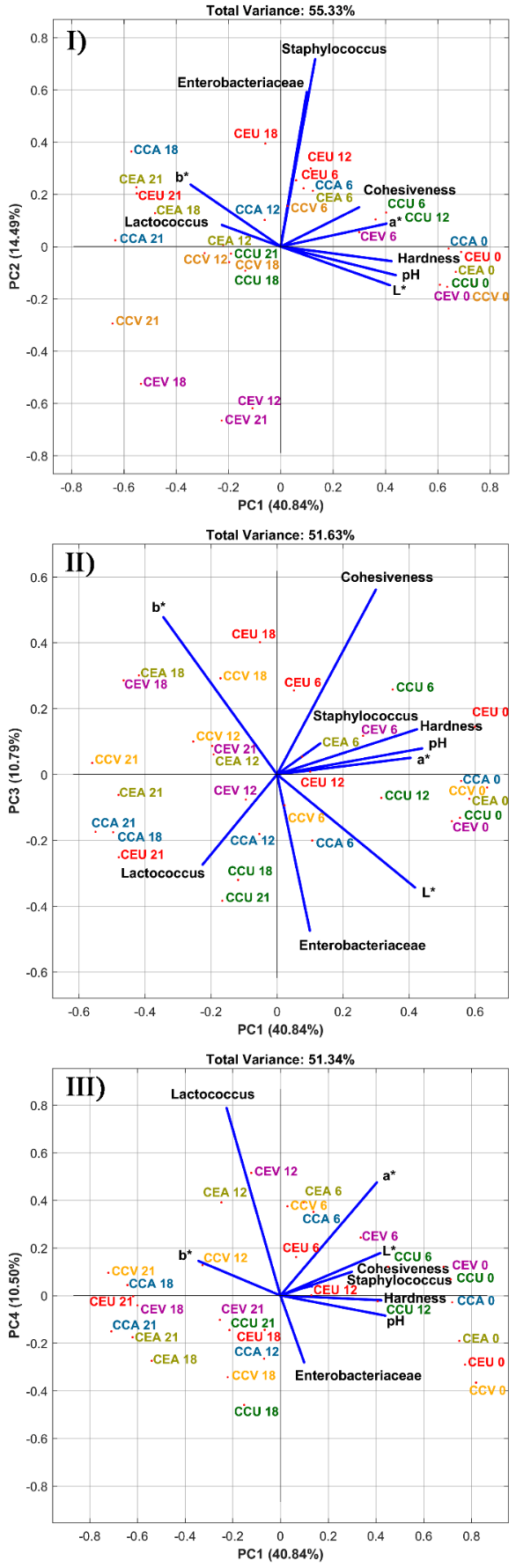


Figure S2. Biplot graphic of the first principal components (77% of accumulated variance) for the physicochemical and microbial quality parameters of raw goat milk cheeses during 21 storage days at 4° C. *L**: lightness; *a**: greenness; *b**: yellowness.
